# Connexin43 Hemichannel-Mediated Regulation of Connexin43

**DOI:** 10.1371/journal.pone.0058057

**Published:** 2013-02-27

**Authors:** Kai Li, Yuan Chi, Kun Gao, Qiaojing Yan, Hiroyuki Matsue, Masayuki Takeda, Masanori Kitamura, Jian Yao

**Affiliations:** 1 Department of Molecular Signaling, Interdisciplinary Graduate School of Medicine and Engineering, University of Yamanashi, Chuo, Yamanashi, Japan; 2 Department of Urology, Interdisciplinary Graduate School of Medicine and Engineering, University of Yamanashi, Chuo, Yamanashi, Japan; 3 Department of Surgical Oncology and General Surgery, First Hospital of China Medical University, Shenyang, China; 4 Department of Dermatology, Chiba University School of Medicine, Chiba, Japan; Baylor College of Medicine, United States of America

## Abstract

**Background:**

Many signaling molecules and pathways that regulate gap junctions (GJs) protein expression and function are, in fact, also controlled by GJs. We, therefore, speculated an existence of the GJ channel-mediated self-regulation of GJs. Using a cell culture model in which nonjunctional connexin43 (Cx43) hemichannels were activated by cadmium (Cd^2+^), we tested this hypothesis.

**Principal Findings:**

Incubation of Cx43-transfected LLC-PK1 cells with Cd^2+^ led to an increased expression of Cx43. This effect of Cd^2+^ was tightly associated with JNK activation. Inhibition of JNK abolished the elevation of Cx43. Further analysis revealed that the changes of JNK and Cx43 were controlled by GSH. Supplement of a membrane-permeable GSH analogue GSH ethyl ester or GSH precursor N-acetyl-cystein abrogated the effects of Cd^2+^ on JNK activation and Cx43 expression. Indeed, Cd^2+^ induced extracellular release of GSH. Blockade of Cx43 hemichannels with heptanol or Cx43 mimetic peptide Gap26 to prevent the efflux of GSH significantly attenuated the Cx43-elevating effects of Cd^2+^.

**Conclusions:**

Collectively, our results thus indicate that Cd^2+^-induced upregulation of Cx43 is through activation of nonjunctional Cx43 hemichannels. Our findings thus support the existence of a hemichannel-mediated self-regulation of Cx43 and provide novel insights into the molecular mechanisms of Cx43 expression and function.

## Introduction

Gap junctions (GJs) are clusters of transmembrane channels that permit the direct intercellular exchange of ions, secondary messengers, and small signaling molecules. Each GJ channel is composed of two hemichannels that reside in the plasma membrane of two closely apposed cells. GJs are formed by a family of special proteins termed connexin (Cx). Among different isoforms of Cx molecules, connexin43 (Cx43) has been extensively investigated because of its ubiquitous expression in almost all cell types [1], [2], [3].

GJs have been implicated in various pathological situations, including those caused by oxidative stress [4], [5], [6], [7]. The majority of the biological effects of GJs are mediated by the direct transmission of signaling molecules among neighboring cells [1], [2], [3]. Besides intercellular GJs channels, the nonjunctional Cx hemichannels also contribute to the regulation of cell function and survival through the extracellular release of the important signaling molecules, such as ATP, NAD(+), GSH, or glutamate [2], [8], [9], [10]. Activation of the hemichannels has been reported in a variety of pathological situations, including those involving oxidative stress [6], [7], [11].

Given the importance of GJs in the control of various cellular processes, regulation of GJs and its forming proteins has been a subject of extensive investigations. Up to now, many signaling molecules have been reported to be able to regulate GJ protein expression and function. Most of them are, in fact, small molecules that can freely pass through GJs or hemichannels, such as Ca^2+^, ATP, IP_3_, ROS and cAMP [1], [2], [3], [4], [5]. In this context, any change in GJ channels or hemichannels should have great influence on the dynamics and whereabouts of these signal molecules, affecting the related signaling pathways and their effects. Along this thinking, one would expect an existence of GJ channel-mediated self-regulation of GJs. However, this hypothesis has never been tested.

Cadmium ion (Cd^2+^) is one of the major metal pollutants, which induces various cell responses through induction of oxidative stress [12], [13]. Cd^2+^ promotes the formation of oxygen free radical [14] and decreases the concentration of the important antioxidant glutathione (GSH) [15], [16]. Modulation of intracellular redox status or inhibition of the stress-related signal such as *c*-Jun N-terminal kinase (JNK) has been documented to attenuate and even prevent Cd^2+^–initiated cell responses, including cell injury [13], [17], [18].

Recently, we have reported that Cx43 hemichannels exaggerated Cd^2+^-elicited cell injury through extracellular efflux of the major antioxidant GSH and subsequent activation of JNK [6]. Because both GSH and JNK have been reported to regulate Cx43 [4], [5], [19], [20], [21], [22], [23], we speculated that the hemichannel opening might also affect Cx43 expression. If so, the hypothesis about the channel-mediated self-regulation of GJs could be validated.

Here, we presented our results showing that Cd^2+^-triggered upregulation of Cx43 was mediated by nonjunctional Cx43 hemichannels. Thus we propose an existence of hemichannel-mediated self-regulation of Cx43.

## Materials and Methods

### Reagents

Cx mimetic peptides Gap20 (sequence EIKKFKYGIEEHC) and Gap26 (sequence VCYDKSFPISHVR) were synthesized at purity of 90% by Invitrogen (Tokyo, Japan). Antibodies against the JNK and *c*-Jun proteins were purchased from Cell Signaling Inc (Beverly, MA, USA). GSH-Glo^TM^ assay kit was purchased from Promega (Madison, WI, USA). Cadmium chloride (CdCl_2_), glutathione reduced ethyl ester (GSHee), N-acetyl-cystein (NAC), SP600125, heptanol, anti-Cx43 and anti-β-actin antibodies, FBS, trypsin/EDTA, antibiotics as well as all other chemicals were obtained from Sigma (Tokyo, Japan).

### Permanent Transfection of LLC-PK1 Cells with Cx43-EGFP

Porcine kidney epithelial cell line LLC-PK1 was purchased from American Type Culture Collection (Rockville, MD). Cx43 pEGFP1 was kindly gifted by Dr. Oyamada (Department of Pathology, Kyoto Prefectural University of Medicine, Kyoto, Japan). The Cx43 pEGFP vector was constructed by ligation of the DraI fragment of rat Cx43 cDNA into the SmaI site of the pEGFP-N1 vector (Clontech, Palo Alto, CA). The vectors were transfected into LLC-PK1 cells by using Lipofectamin Plus reagent (Invitrogen, Carlsbad, USA), following the manufacturer's instruction [6]. To obtain LLC-PK1 cell clones stably expressing Cx43-EGFP, the selective medium containing 200 μg/ml G418 was added into the culture and renewed at the 4-day intervals. Clones with high levels of GFP were selected under fluorescence microscope and used for this study.

### Transient Transfection of LLC-PK1 Cells with a Wild-type Cx43

LLC-PK1 cells were transiently transfected with pMSCV-Cx43 vector (kindly provided by Dr. Walter Eckhart, The Salk Institute for Biological Studies, San Diego) or pEGFP-N1 using gene juice (Novagen, Merck, Germany). After 36 h, cells were either left untreated or exposed to Cd2+ for the indicated time. Cellular protein was extracted and subjected to the Western blot analysis. The transfection efficiency was estimated based on the number of cells expressing green fluorescent protein under immunofluorescence microscopy.

### Western Blot Analysis

Western blot was performed by the enhanced chemiluminescence system [6], [24]. Briefly, equal amounts of extracted cellular proteins were separated by 10% SDS–polyacrylamide gels and electrotransferred onto polyvinylidene difluoride membranes. After blocking with 3% BSA in PBS, the membranes were incubated with primary antibody. After washing with PBS-0.1% Tween 20, filters were probed with horseradish peroxidase–conjugated sheep anti-rabbit IgG or rabbit anti-mouse IgG. Immunoreactivity was detected by the enhanced chemiluminescence system (Amersham Biosciences, Buckinghamshire, UK). The chemiluminescent signal is captured with a Fujifilm luminescent image LAS-4000 analyzer (Fujifilm, Tokyo, Japan). To confirm the equal loading per lane, filters were treated with 2% SDS and 100 mM β-mercaptoethanol in 62.5 mM Tris-HCl (pH 6.8) for 30 min at 60°C and reprobed for β-actin. Data shown are representative of at least three independent experiments with similar results.

### ATP Measurement

ATP was measured using a luciferin/luciferase bioluminescence assay kit (Molecular Probes). The intensity of chemiluminescent signal was determined by a luminometer (Gene Light 55; Microtech Nition, Chiba, Japan) as described before [6], [25].

### GSH Measurement

GSH activity was measured by using GSH-Glo^TM^ assay kit Promega (Madison, WI, USA) [6]. Briefly, cells at confluent culture in 96-well culture plates were exposed to 35 μM Cd^2+^ for 6 h. The supernatants were collected for GSH measurement according to the protocols provided by the manufacturer.

### Cytotoxicity Assay

Cytotoxicity was evaluated by the release of lactate dehydrogenase (LDH) using an LDH cytotoxicity detection kit (Takara Bio Inc., Otsu, Shiga, Japan), as described previously [6].

### Statistical analysis

Values are expressed as mean ± SD or SE. Comparison of two populations was made using Student's *t*-test. For multiple comparisons with a single control, one-way analysis of variance (ANOVA) followed by Dunnett's test was employed. Both analyses were carried out using SigmaStat statistical software (Jandel Scientific, CA, USA). *P*<0.05 was considered to be a statistically significant difference.

## Results

### Cd^2+^ stimulates Cx43 expression in Cx43-LLC-PK1 cells

Our previous studies demonstrated that porcine proximal tubular epithelial cell line LLC-PK1 did not express Cx43 nor had functional gap junctional intercellular communication [26], [27]. Transfection of LLC-PK1 cells with a Cx43-EGFP gene markedly enhanced their susceptibility to the cytotoxic effect of Cd^2+^ through hemichannel-mediated loss of GSH [6]. Using the same cell model, we examined the influence of Cx43 hemichannels on Cx43 expression.

**Figure 1:**
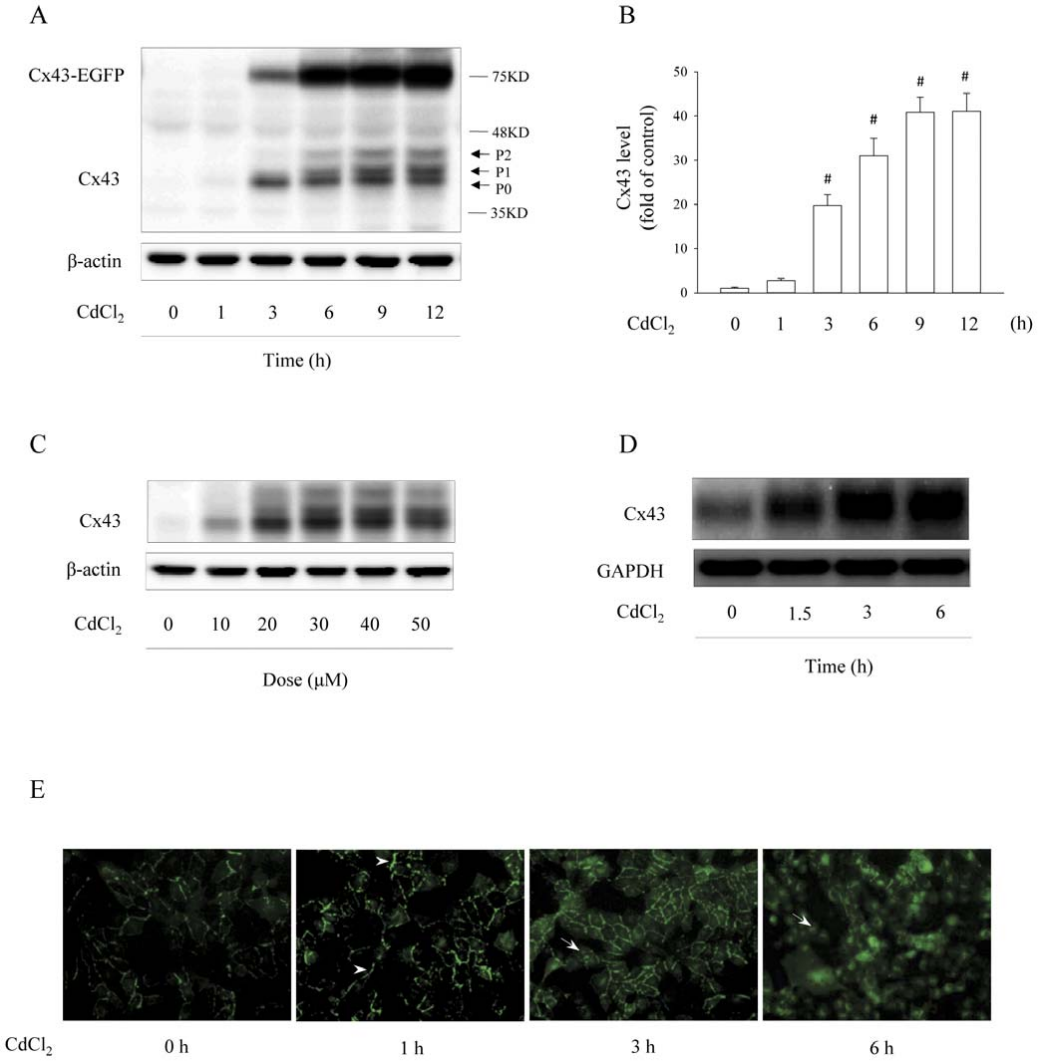
Effects of Cd^2+^ on Cx43 expression in Cx43-LLC-PK1 cells. (A–C) Time- and dose-dependent effects of Cd^2+^ on Cx43 protein levels. Cx43-LLC-PK1 cells were treated with 35 μM CdCl_2_ for the indicated duration (A) or exposed to the indicated concentrations of CdCl_2_ for 6 h (C). The cellular protein was extracted and subjected to Western blot analysis of Cx43. In Western blot (A), Cx43 was detected at the molecular near 70 kDa (upper band) and 43 kDa, representing EGFP-tagged and untagged Cx43, respectively. P0, P1 and P2 denoted the nonphophorylated, phosphorylated and hyper-phosphorylated Cx43. (B) Densitometric analysis of Cx43 expression shown in A. Results were expressed as induction relative to the basal level of Cx43 (mean ± S.D., n = 3). # *p*<0.01 versus untreated control. (D) Effect of Cd^2+^ on Cx43 mRNA expression. Cx43-LLC-PK1 cells were exposed to 35 μM CdCl_2_ for the indicated duration. Cellular RNA was extracted and subjected to Northen blot analysis of Cx43. The level of GAPDH was shown as a loading control. (E) Effect of Cd^2+^ on Cx43-EGFP distribution. LLC-PK1 cells permanently transfected with a vector encoding Cx43-EGFP were exposed to 35 μM CdCl_2_. The expression and localization of Cx43-EGFP at different time points following Cd^2+^ stimulation were shown. Note the obviously increased expression of Cx43-EGFP and shift of fusion protein from cell membrane (arrow head) to perinuclear region (white arrow) after incubation with Cd^2+^.

Western blot analysis of the cellular lysate from Cx43-LLC-PK1 cells revealed that Cx43 protein existed at two forms: one was the EGFP-tagged Cx43, localized at the location of about 70 kDa; the other was the untagged Cx43, probably released by proteolysis of Cx43-EGFP [28], localized at near 43 kDa (Fig. 1A). Exposure of Cx43-LLC-PK1 cells to Cd^2+^ resulted in a time-dependent elevation in both EGFP-tagged and untagged Cx43 (Fig. 1B, Fig. S1). Both forms were, in fact, increased in a paralleled way. However, the untagged Cx43 displayed an even more sensitive response to Cd^2+^_,_ as manifested by the appearance of two characteristic slowly migrating bands, representing the phosphorylated (*P*1) and hyperphosphorylated forms (*P*2) of Cx43. For this reason, the following experiments focused on changes in the untagged Cx43.

Dose response analysis revealed that the effect of Cd^2+^ on Cx43 was concentration-dependent. Cd^2+^ at the concentration of 35 μM markedly increased Cx43 protein expression at both phosphorylated and nonphosphorylated forms (Fig. 1C). Northern blot analysis revealed that Cd^2+^ also caused a rapid elevation in Cx43 mRNA levels (Fig. 1D). These results indicate that Cd^2+^ induces Cx43 expression at both protein and mRNA levels. In addition, it also promotes posttranslational modification of Cx43.

### Cd^2+^ alters Cx43 localization

LLC-PK1 cells positively transfected with Cx43-EGFP showed linear localization of the fusion protein on the plasma membrane between adjacent cells (Fig. 1E). Exposure of these cells to Cd^2+^ for 3 h resulted in an obvious increase in GFP at the region of cell-to-cell contacts and an appearance of GFP at perinuclear region. At 6 h, most of the cells became smaller and round-shaped. The fusion protein largely disappeared from plasma membrane. Instead, there was strong GFP at the perinuclear region. IF staining of Cx43 confirmed that Cx43 protein was co-localized with GFP and displayed similar changes as GFP in response to Cd^2+^ stimulation (Fig. S2). These results indicate that Cd^2+^ induces Cx43 expression and causes intracellular localization of Cx43.

### JNK mediates Cd^2+^-elicited Cx43 expression

**Figure 2:**
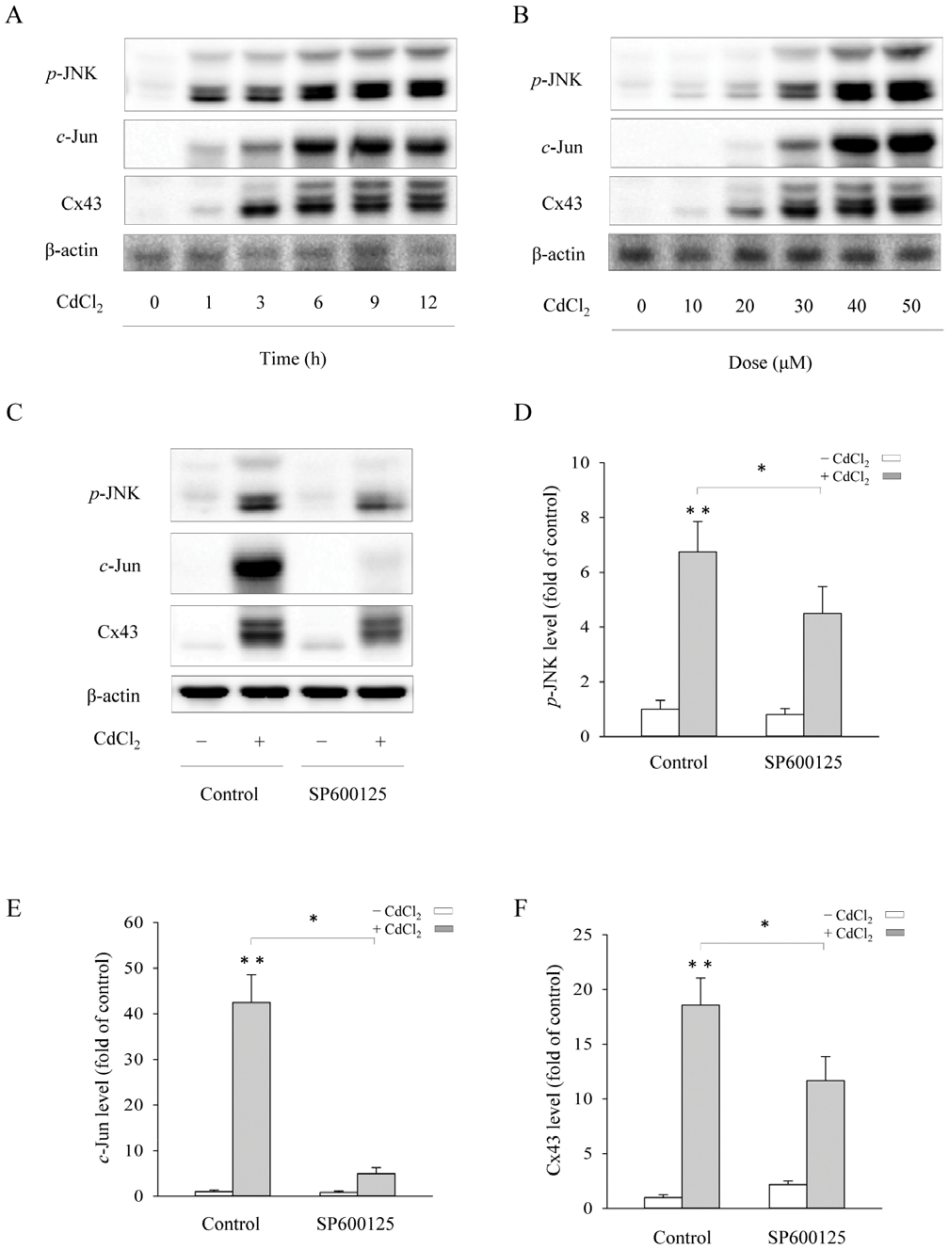
Involvement of JNK activation in the induction of Cx43 protein by Cd^2+^. (A–B) Cd^2+^-induced *p*-JNK and *c*-Jun activation. LLC-PK1 cells were exposed to 35 μM Cd^2+^ for the indicated time (A) or various concentrations of Cd^2+^ (B) for 6 h. The cellular protein was extracted and subjected to Western blot analysis of phosphorylated (*p*) JNK, *c*-Jun and Cx43. The level of β-actin was taken as loading control. (C–F) Suppression of Cd^2+^-elicited increase of *p*-JNK, *c*-Jun and Cx43 levels by a JNK inhibitor. LLC-PK1 cells were pretreated with 50 μM SP600125 for 30 min before exposing them to 35 μM Cd^2+^ for 6 h. Cellular proteins were analyzed for *p*-JNK, *c*-Jun, Cx43 and β-actin (C). (D–F) Densitometric analysis of *p*-JNK, *c*-Jun and Cx43 expression shown in C. Results were expressed as relative induction compared with the basal level (mean ± S.D., n = 3). * *p*<0.05 versus Cd^2+^ alone, and ** <0.01 versus untreated control.

As a stress-activated kinase, JNK mediates Cd^2+^-initiated cell injury [6], [13], [17], [18]. In addition, one of the downstream molecules of JNK, *c*-Jun, is known to be able to induce Cx43 gene expression [29], [30]. We therefore assessed the role of JNK activation in Cd^2+^-elicited elevation of Cx43. As shown in Fig. 2A, 2B, Cd^2+^ induced elevation of Cx43 was tightly associated with JNK activation, as revealed by the increased phosphorylation of JNK (*p*-JNK). Inhibition of JNK with the specific inhibitor SP600125 largely abrogated the effect of Cd^2+^ on Cx43 (Fig. 2C, 2F). The effectiveness of the inhibitor on JNK and *c*-Jun was shown in Fig. 2C–2E. These results suggest that JNK mediates the Cd^2+^-elicited upregulation of Cx43.

### Supplement of exogenous GSH abrogates the effect of Cd^2+^ on Cx43 and JNK activation

**Figure 3:**
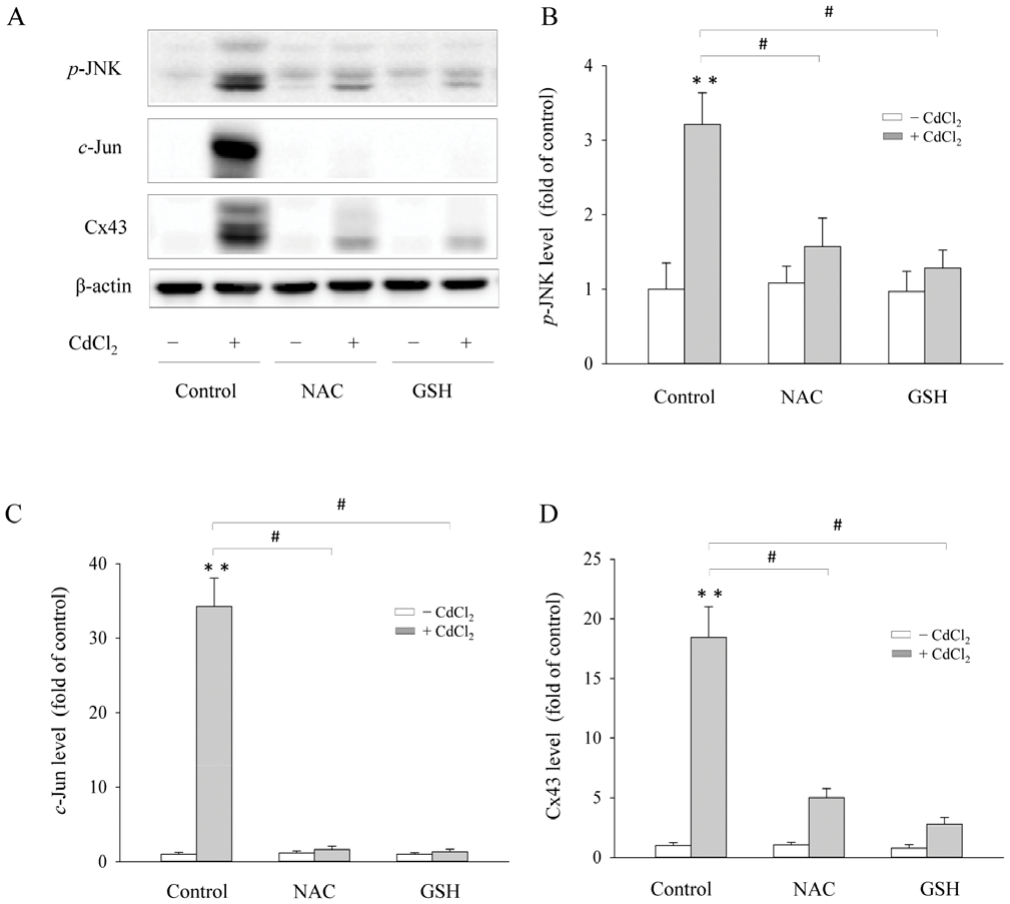
Effects of GSH-upregulating agents on Cd^2+^-elicited Cx43 expression. Cx43-LLC-PK1 cells were pretreated with 2 mM GSHee or 2 mM NAC for 1 h and exposed to 35 μM Cd^2+^ for additional 6 h. Cellular protein was subjected to Western blot analysis for *p*-JNK, *c*-Jun, Cx43 and β-actin (A). Densitometric results (B–D) were expressed as induction relative to the basal level of *p*-JNK, *c*-Jun and Cx43, respectively (mean ± S.D., n = 3). # *p*<0.01 versus Cd^2+^ alone, and ** <0.01 versus untreated control.

Previous studies indicated that Cd^2+^-triggered JNK activation was controlled by the intracellular GSH concentrations [6], [31]. We, therefore, tested whether the Cx43 expression caused by Cd^2+^ could also be influenced by GSH. As shown in Fig. 3, supplement of cells with a GSH precursor NAC [32] or direct addition of a membrane-permeable GSH analogue GSHee into culture significantly attenuated JNK activation and *c*-Jun level (Fig. 3A–3C). Concomitantly, Cx43 expression was also reduced (Fig. 3A, 3D). These results thus indicate a key role of intracellular GSH in Cd^2+^-triggered activation of JNK and elevation of Cx43.

### Inhibition of Cx43 hemichannels attenuates the Cd^2+^-elicited Cx43 expression and JNK activation

**Figure 4:**
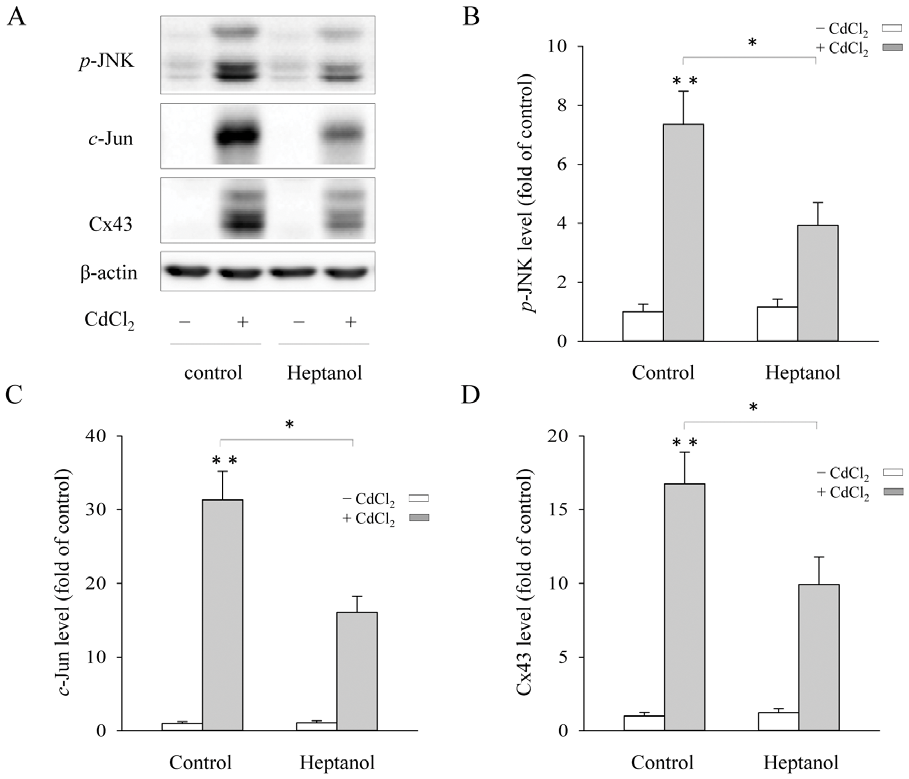
Influence of gap junctions (GJs) blocker on Cx43 expression and JNK activation. (A) Cx43-LLC-PK1 cells were pretreated with GJs blocker, 4 mM heptanol for 30 min before exposing them to 35 μM Cd^2+^ for 6 h. Cellular proteins were analyzed for *p*-JNK, *c*-Jun, Cx43 and β-actin. (B–D) Densitometric analysis of *p*-JNK, *c*-Jun and Cx43 expression shown in A. Results were expressed as relative induction compared with the basal level (mean ± S.D., n = 3). * *p*<0.05 versus Cd^2+^ alone, and ** <0.01 versus untreated control.

Given that opening of Cx43 hemichannels causes efflux of GSH [6], [9], we, therefore, asked whether blockade of hemichannels could also affect Cd^2+^-elicited Cx43 expression and JNK activation. As shown in Fig. 4, GJ blocker heptanol significantly decreased Cd^2+^-elicited Cx43 expression and JNK activation.

**Figure 5:**
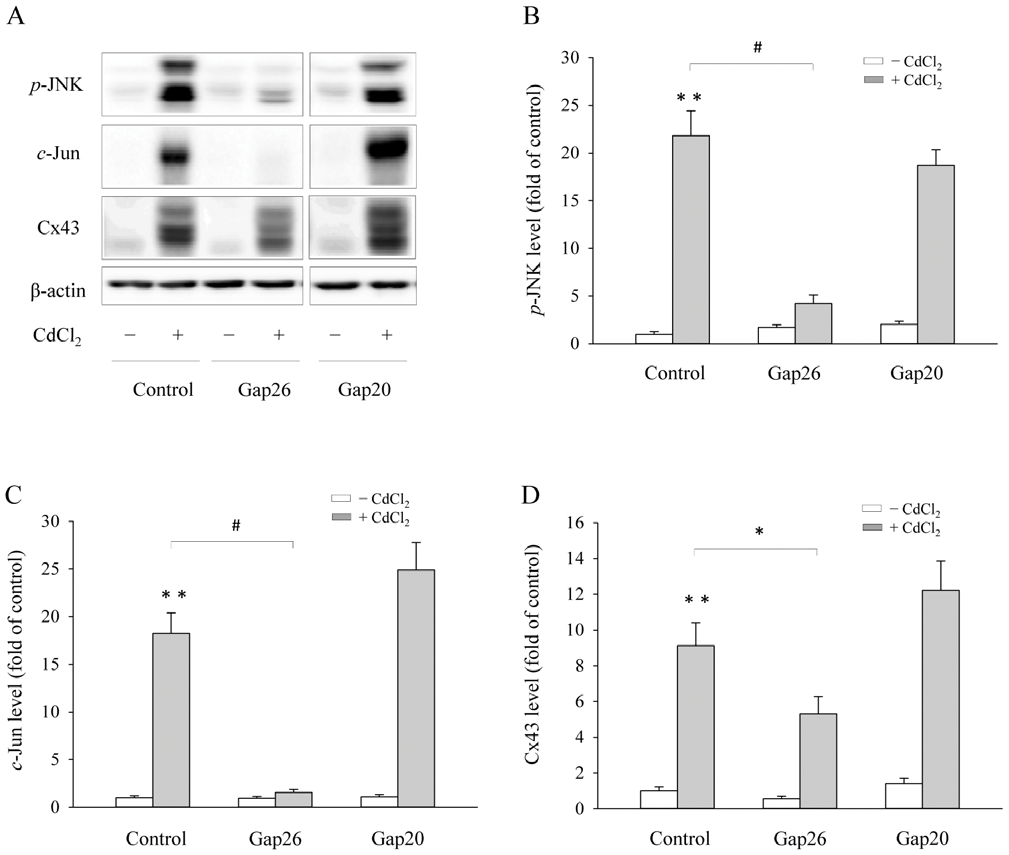
Effects of various connexin (Cx) mimetic peptides on Cx43 and *p*-JNK, *c*-Jun levels. (A) Cx43-LLC-PK1 cells were pretreated with various Cx mimetic peptides, Gap20, and Gap26 at the concentration of 100 μM for 1 h, and then exposed to 35 μM Cd^2+^ in the presence of the peptides for additional 6 h. Cellular protein was subjected to Western blot analysis for *p*-JNK, *c*-Jun, Cx43 and β-actin. Densitometric results (B–D) were expressed as induction relative to the basal level of *p*-JNK, *c*-Jun and Cx43, respectively (mean ± S.D., n = 3). # *p*<0.01 and * *p*<0.05 versus Cd^2+^ alone, and ** <0.01 versus untreated control.

To further establish the role of Cx43 hemichannels, we used Cx43 mimetic peptide Gap26, which is known to specifically block Cx43 hemichannels without interference on intercellular communication [9], [33]. As shown in Fig. 5, Gap26 effectively prevented Cd^2+^-elicited JNK activation and Cx43 expression (Fig. 5). As a control, a peptide Gap20, a synthetic peptide corresponding to a sequence in the intracellular loop of Cx43, was ineffective.

**Figure 6:**
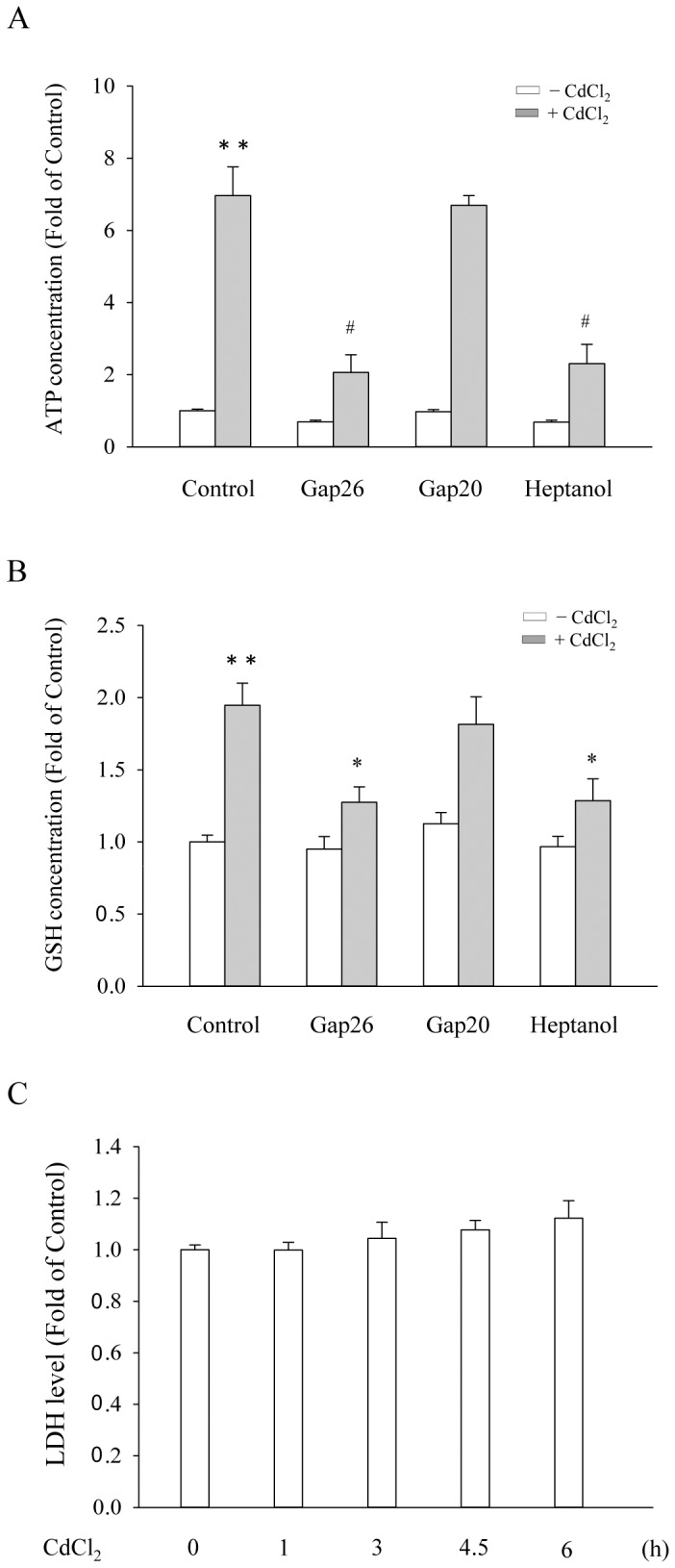
Blockade of Cd^2+^-induced ATP and GSH release by GJs inhibitor and Cx mimetic peptides. (A–B) Cx43-LLC-PK1 cells were pretreated with 4 mM heptanol for 30 min or with Cx43 mimetic peptides Gap20 and Gap26 at the concentration of 100 μM for 1 h, and then exposed to 35 μM Cd^2+^ in the presence of the pretreated agents for additional 6 h ATP and GSH concentration in culture medium was measured. The data were expressed as the fold induction against basal level (mean ± S.E., n = 4). # *p*<0.01 and * *p*<0.05 versus Cd^2+^ alone, and ** <0.01 versus untreated control. (C) Time course effects of Cd^2+^ on LDH release. Cx43-LLC-PK1 cells were treated with 35 μM CdCl_2_ for the indicated duration. The supernatants were collected and assayed for LDH activity. The data were expressed as fold of zero point control (mean ± S.E., n = 4).

To confirm the opening of Cx43 hemichannels and induction of GSH release by Cd^2+^, we measured the extracellular ATP and GSH concentrations in the presence of or absence of the specific hemichannel-interacting peptide Gap26. As shown in Fig. 6, Cd^2+^ induced the extracellular release of ATP and GSH. This effect was significantly blocked by Gap26, but not by Gap20. In addition, a nonspecific GJ channel/hemichannel inhibitor heptanol also abrogated this effect. Of note, incubation of cells with 35 μM Cd^2+^ for 6 h did not greatly influence cell viability, as evaluated by the release of LDH (Fig. 6C).

These observations thus indicate that Cx43 hemichannel opening contributes to Cd^2+^-elicited elevation of Cx43.

### Cd^2+^ stimulates Cx43 expression in the mixed cultures containing both Cx43-positive and Cx43-null LLC-PK1 cells

**Figure 7:**
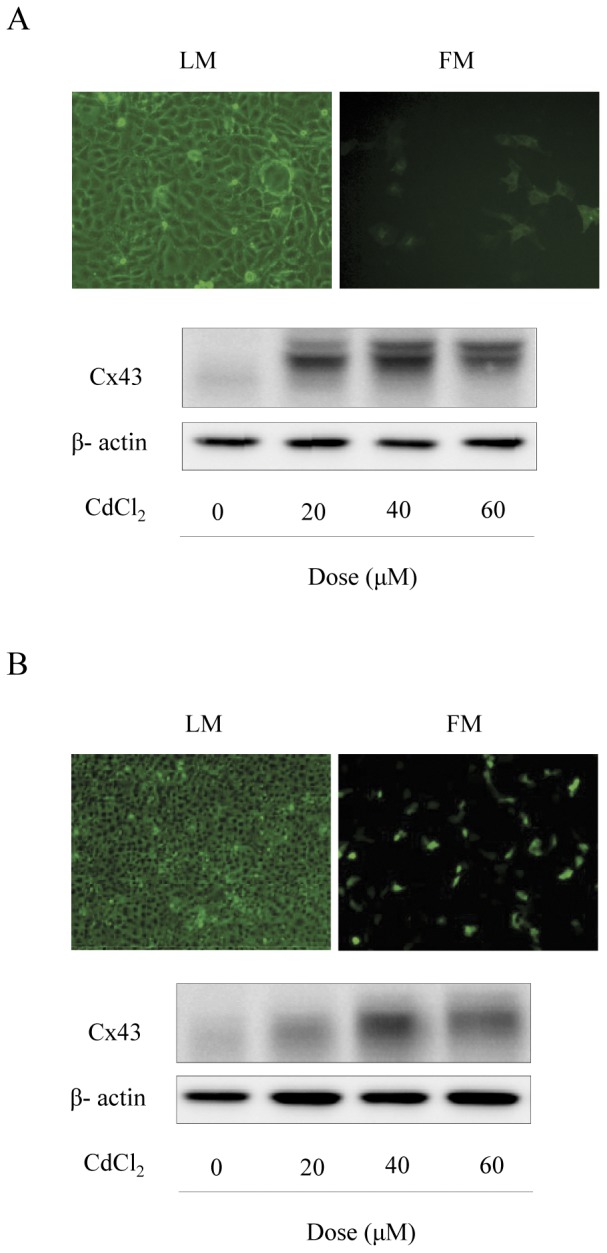
Effects of Cd^2+^ on Cx43 expression in the mixed culture containing both Cx43-positive and Cx43-null LLC-PK1 cells. (A) Cx43-LLC-PK1 cells and LLC-PK1 cells were cultured in 1∶4 proportions. The number and localization of Cx43-EGFP positive cells under fluorescent microscope in the mixed culture is shown (upper panel, images under light and fluorescent microscope, x200). The mixed culture was exposed to the indicated concentrations of CdCl_2_ for 6 h. The cellular protein was extracted and subjected to Western blot analysis of Cx43 (lower panel). (B) LLC-PK1 cells were transiently transfected with a wild-type Cx43 gene or EGFP1 vector for 36 h. The number and distribution of EGFP-positive and negative cells under fluorescent microscope are shown and used for estimation of transfection efficiency by comparing with total cells under light microscope and fluorescent microscope (upper panel). The cells transiently transfected with a wild-type Cx43 gene were exposed to the indicated concentrations of CdCl_2_ for 6 h. Cellular protein was subjected to Western blot analysis for Cx43 (lower panel).

Given that Gap26 has also been reported to be able to disrupt gap junctional intercellular communication [9], [33], we therefore tried to confirm or exclude the possible involvement of GJs in Cd^2+^-induced elevation of Cx43. Toward this objective, we have co-cultured Cx43-EGFP LLC-PK cells with Cx43-null LLC-PK1 cells in a ratio of 1 to 4. Under this condition, GFP-positive Cx43 cells were isolated from each other with little possibility to form functional GJs (Fig. 7A, upper panel). Interestingly, Cd^2+^ still stimulated Cx43 expression (Fig. 7A, lower panel). This result thus indicates that GJ is not a prerequisite for the Cx43-elevating effect of Cd^2+^.

To exclude the possible influence of EGFP, we also examined the effect of Cd^2+^ in LLC-PK1 cells transiently transfected with a wild-type Cx43 gene without EGFP. The transfection efficiency as estimated from control cells transfected with a pEGFP-N1 vector. Based on the number of EGFP-positive and negative cells under the fluorescence microscopy (Fig. 7B, upper panel), transfection rate was less than 30%. However, even under such a condition, Cd^2+^ still stimulated Cx43 expression and promoted Cx43 phosphorylation, as revealed the upward shift of Cx43 bands (Fig. 7B, lower panel). This result also did not support a critical involvement of GJs in the effect of Cd^2+^. It also excluded the possible interference of the tagged EGFP on the assay system.

## Discussion

**Figure 8:**
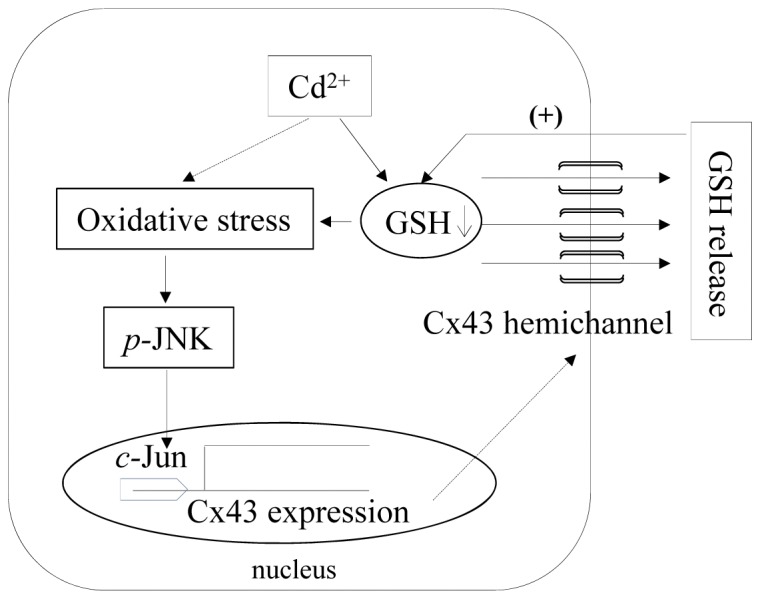
Schematic diagram illustrating Cx43 hemichannel-mediated self-regulation of Cx43 under the stimulation of Cd^2+^. Induction of oxidative stress and depletion of GSH by Cd^2+^ results in the opening of Cx43-hemichannels, leading to the efflux of the major antioxidant GSH. The loss of GSH exaggerates oxidative stress, promoting JNK activation and JNK-mediated Cx43 mRNA expression. The increased synthesis of Cx43 might enhance the formation of nonjunctional hemichannels, further worsening the loss of GSH. The vicious autoregulation loop between GSH and hemichannels provides a molecular mechanism for the hemichannel-mediated regulation of Cx43.

In this study, we revealed a presently unrecognized regulatory mechanism of Cx43 expression. The molecular events involved are schematically depicted in Fig. 8. Given the importance of Cx43 in the control of cell behaviors, our finding may open a new window toward our further understanding of the regulatory mechanisms and roles of Cx43 in various pathophysiological situations.

Exposure of Cx43-LLC-PK1 cells to Cd^2+^ led to an increased expression of Cx43. This effect of Cd^2+^ was similarly blocked by supplement of GSH, inhibition of JNK or dysfunction of Cx43 hemichannels, indicating that these molecular events were closely interlinked. Consistent with this thinking, supplement of GSH blocked the JNK activation, while inhibition of JNK abolished the elevation of Cx43. It is concluded that GSH-JNK signaling cascade underlies the elevated Cx43. This conclusion is perhaps not surprising, since the central roles of GSH and JNK in Cd^2+^-induced cell responses have been previously documented [6], [13], [17], [18]. In addition, there are also reports describing an involvement of GSH and JNK in the regulation of Cx43 [4], [19], [20], [21], [22], [23].

What is interesting, however, is that Cd^2+^-induced elevation of Cx43 could also be significantly blocked by inhibition of Cx43-hemichannels. In other words, Cx43 was regulated by its forming channels. What was the signaling mechanism behind the self-regulation? In our previous report, we have demonstrated that the opening of Cx43 hemichannels by Cd^2+^ was a downstream event of the reduced intracellular GSH and JNK activation, as evidenced by the observations that supplement of GSH or inhibition of JNK significantly prevented Cd^2+^-induced release of ATP (an indicator of hemichannel opening). Reciprocally, we found that the opening of Cx43-hemichannels exaggerated the reduction of intracellular GSH and activation of JNK. Our data thus pointed to existence of “a positive autoregulation loop” between the decreased GSH and hemichannel opening in cell response to Cd^2+^. In this loop, the initial depletion of GSH by Cd^2+^ resulted in activation of JNK, causing the opening of Cx43 hemichannels; whereas the opening of Cx43 hemichannels led to further loss of GSH, thus forming “a vicious cycle” that advanced Cd^2+^-elicited oxidative stress and cell injury. Our proposed model of “the positive regulation loop” also explains the observed self-regulation of Cx43.

How did the regulation loop affect Cx43 expression and phosphorylation? We think that the increased Cx43 could be due to the c-Jun-mediated activation of Cx43 gene. The presence of AP-1 binding site in the promoter region of Cx43 gene has been previously reported [21], [30]. Indeed, Cd^2+^ also induced a marked increase in Cx43 mRNA expression in this study. Interestingly, as a downstream target of JNK, c-Jun was also controlled by the regulation loop. Regarding the increased phosphorylation of Cx43, it could be related to the oxidative stress-activated kinases, such as MAPK. Involvement of these kinases in phosphorylating Cx43 has been extensively documented [1], [5]. Moreover, these kinases should be subjected to the control of the regulatory loop in a way similar to JNK.

Of note, the evidences indicating the opening of nonjunctional Cx43-hemichannels by Cd^2+^ have been presented in our previous report [6]. They included the Cd^2+^-triggered influx of Lucifer yellow and efflux of ATP. Here, we provided additional evidence supporting the opening of Cx43 hemichannels by Cd^2+^. Gap 26, a Cx43 mimetic peptide that blocks Cx43 hemichannels [9], [33], significantly abrogated the effect of Cd^2+^ on ATP and GSH, whereas control peptide Gap20 had no effect. In a well described model of hemichannel opening induced by lowering extracellular calcium [9], [34], the efflux of ATP and GSH was also blocked by Gap26, but not Gap20 (Fig. S3). In further support of the role of Cx43-hemichannels, we demonstrated that Cd^2+^ similarly stimulated Cx43 expression in the mixed culture system in which Cx43-positive LLC-PK1 cells were surrounded by Cx43-null LLC-PK1 cells and had little possibility to form functional intercellular communication. Therefore, we concluded a critical involvement of Cx43-hemichannels in Cd^2+^-elicited elevation of Cx43.

It is also interesting to mention that incubation of Cx43-EGFP cells with Cd^2+^ for 6 h caused the dominant localization of Cx43 in the perinulcear region. This reasons for the redistribution of Cx43 distribution could be multiple. First, Cd^2+^ was able to induce dissemble of tight junctions [35]. Most of cells at 6 h time point were, in fact, round-shaped and loosely contacted with neighboring cells. Under such a condition, the membrane proteins such as ZO-1, occludin and cadherin were internalized and degraded [36]. Cx43, as a membrane protein closely interacting with these tight junction proteins [37], might also be simultaneously internalized. This may explain the disappearance of the membrane-associated Cx43. Second, our study demonstrated that Cd^2+^ markedly promoted mRNA expression of Cx43. The newly synthesized Cx43 protein was reported to be localized in the perinuclear regions and transported to the plasma membrane through the secretory pathway [38], [39]. Cd^2+^ induced disruption of cytoskeleton and motor protein might block Cx43 traffic from the endoplasmic reticulum to the plasma membrane [38], [40], [41], [42], causing the perinuclear accumulation of Cx43. In addition, the Cd^2+^-induced Cx43 internalization might also contribute to the intracellular localization of Cx43. Intriguingly, the intracellular localization of Cx43 after long-term exposure to Cd^2+^ may be a self-defense mechanism against Cd^2+^-induced oxidative stress and cell injury. Recently, we demonstrated that the hemichannel-mediated loss of GSH exaggerated Cd^2+^-induced oxidative stress and cell injury [6]. The intracellular redistribution of Cx43 would prevent formation and opening of hemichannels, thus preventing the cells from further damage.

Of note, in this study, we have used Gap20 as a peptide control. Consistent with several previous reports [43], [44], [45], we did not detect any influence of Gap20 on nonjunctional hemichannel activity. However, several recent studies indicate an inhibitory effect of L2 domain peptides on hemichannel functions [46], [47]. The question occurs as to why Gap20, which has a sequence corresponding to intracellular L2 domain of Cx43, had no effect. We think that the discrepancy could be explained by the fact that, different from the reported L2 domain peptides, Gap20 was not coupled with the membrane-penetrating motif TAT. It cannot enter the cell under normal culture condition. Consequently, there is little possibility that Gap20 affects hemichannel activity through interaction with intracellular domains.

Collectively, we demonstrated that Cd^2+^-induced elevation of Cx43 is mediated by its forming hemichannels. Given that the Cx43-forming channels (either intercellular GJ channels or hemichannels) transmit multiple signal molecules, including those critically involved in the regulation of Cx43 itself, Cx43 self-regulation may be a common phenomenon in various pathological situations. It may contribute to the initiation or development of multiple cellular processes. Thus, intercepting the autoregulation loop through modification of connexins could be an effective way to change the outcomes of these cellular processes.

## Supporting Information

Figure S1 **Densitometric analysis of time-course effect of cadmium on Cx43-EGFP (A) and phosphorylated Cx43 levels (B) shown in** **Fig. 1A** **.** Results were expressed as fold of induction relative to the basal level of Cx43-EGFP in Fig. A (mean ± S.D., n = 3). # *p*<0.01 versus untreated control. In Fig. B, results were expressed as fold of induction relative to the phosphorylated level of Cx43 at 1 h because of the lack of visible phosphorylated band at zero point (mean ± S.D., n = 3). # *p*<0.01. Note the parallel relationship between EGFP-tagged and untagged Cx43 shown in Fig. 1B, and between total untagged Cx43 and phosphorylated Cx43.
(TIF)

Figure S2 **Immunofluorescent staining of Cx43 in Cx43-EGFP LLC-PK1 cells.** Cx43-EGFP cells were exposed to 35 μM CdCl_2_ for the indicated time and stained for Cx43 (red) and DAPI (nuclei; blue). Immuofluorescent images of EGFP (green), Cx43 (red) and nuclei (blue) were captured using a CCD camera attached to the IF microscope. The merged images of Cx43 and EGFP (yellow) are shown in the right panel. Note the complete overlapping of Cx43 and EGFP (yellow) and the increased intensity of perinuclear Cx43 following Cd^2+^ stimulation.
(TIF)

Figure S3 **Effect of Cx43 mimetic peptides on calcium deprivation-triggered release of ATP and GSH in Cx43-LLC-PK1 cells.** Cx43-LLC-PK1 cells were pretreated with Cx mimetic peptides Gap20 and Gap26 at the concentration of 100 μM for 1 h, and then changed to calcium-free medium containing the same amount of peptides for additional 15 min. ATP (A) and GSH (B) concentration in culture medium was measured. The data were expressed as the fold induction against untreated control (mean ± S.E., n = 4). # *p*<0.01, * *p*<0.05 versus Cd^2+^ alone, and ** <0.01 versus untreated control.
(TIF)
