# Supplementary figures and images for: Connexin43 Hemichannel-Mediated Regulation of Connexin43
Source: PLoS One. 2013 Feb 27;8(2):e58057. doi: 10.1371/journal.pone.0058057 (PMC3584027; doi:10.1371/journal.pone.0058057)

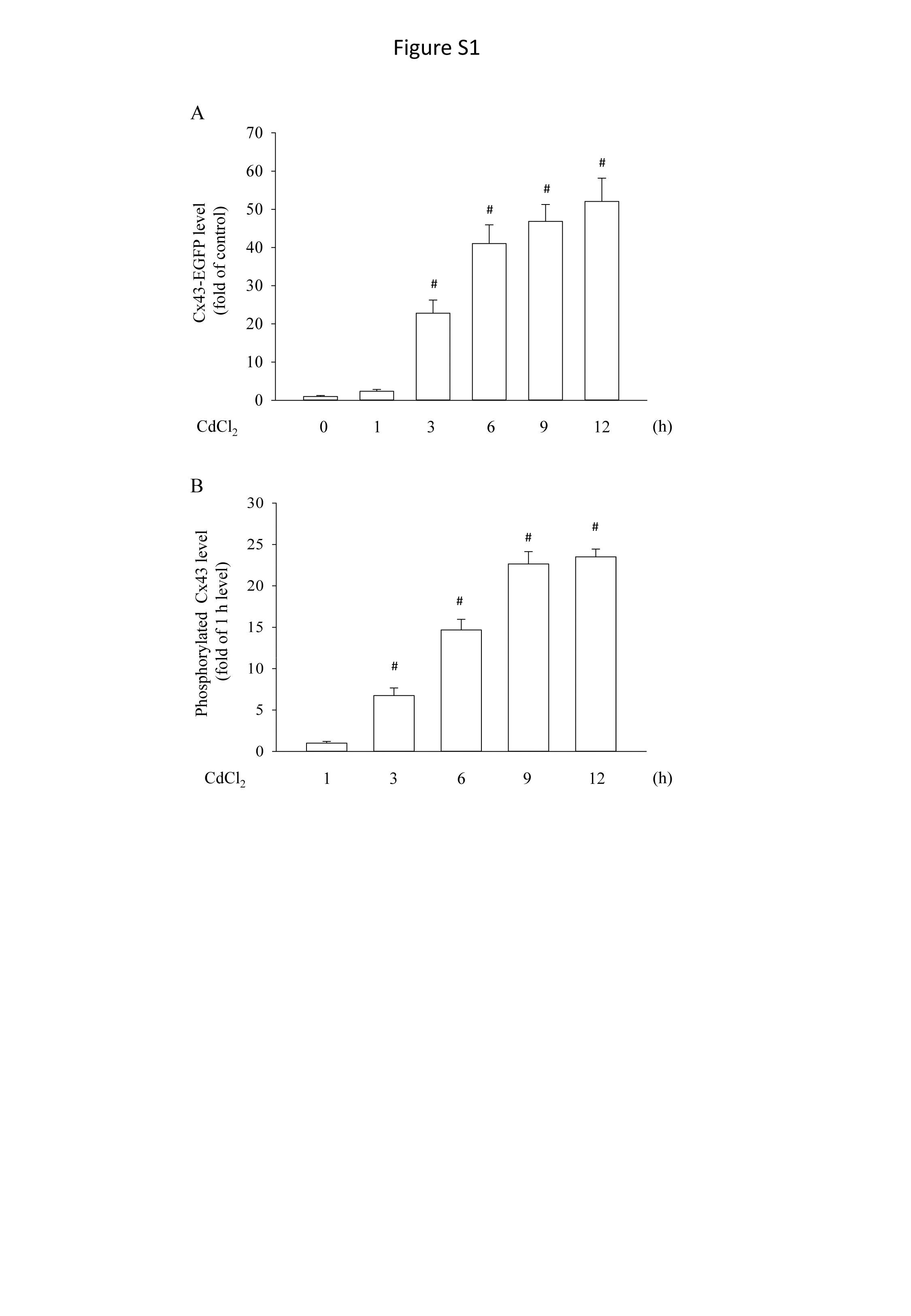

Supplement: Figure S1 — Densitometric analysis of time-course effect of cadmium on Cx43-EGFP (A) and phosphorylated Cx43 levels (B) shown in Fig. 1A . Results were expressed as fold of induction relative to the basal level of Cx43-EGFP in Fig. A (mean ± S.D., n = 3). # p<0.01 versus untreated control. In Fig. B, results were expressed as fold of induction relative to the phosphorylated level of Cx43 at 1 h because of the lack of visible phosphorylated band at zero point (mean ± S.D., n = 3). # p<0.01. Note the parallel relationship between EGFP-tagged and untagged Cx43 shown in Fig. 1B, and between total untagged Cx43 and phosphorylated Cx43. (TIF) [file pone.0058057.s001.tif]

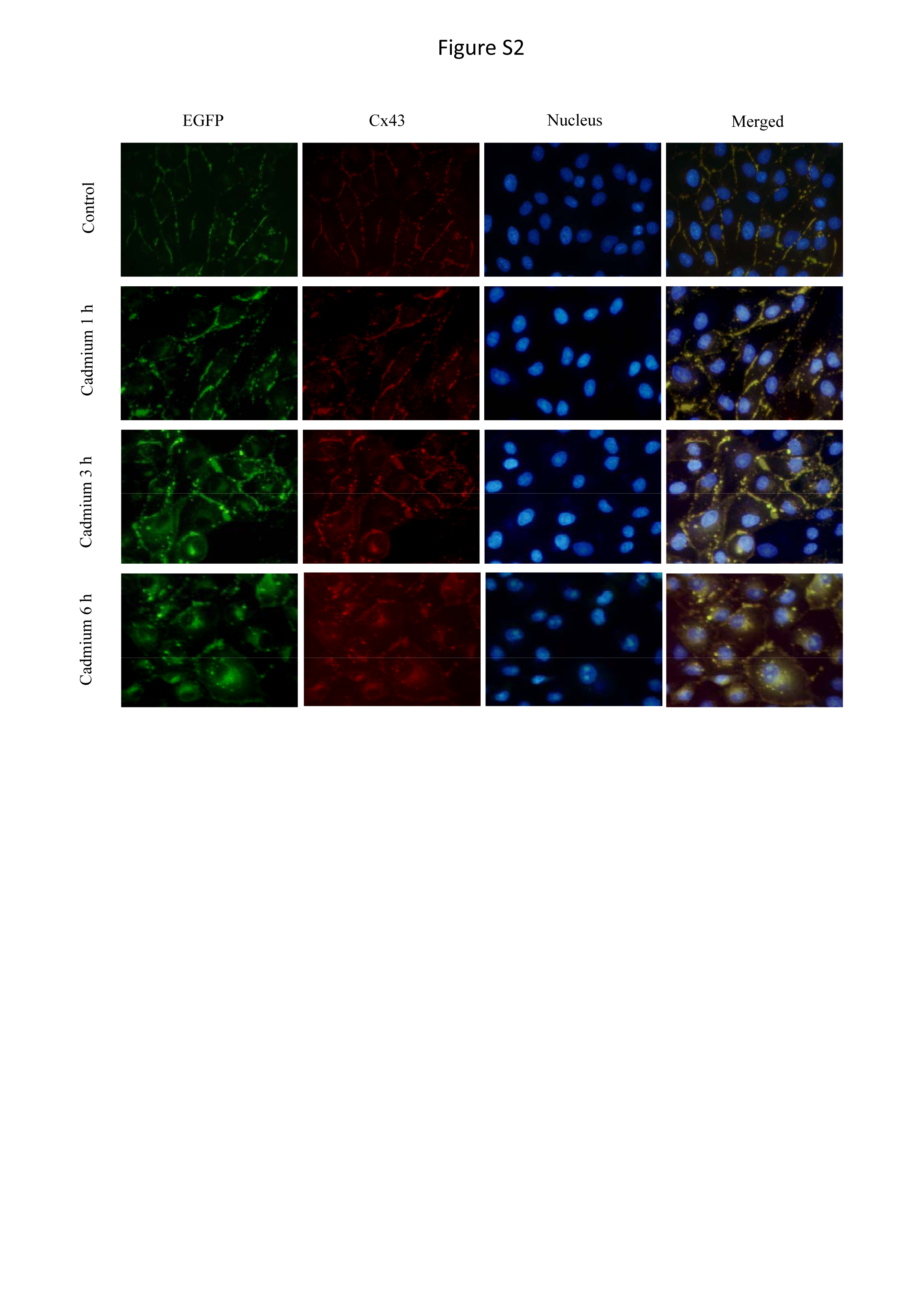

Supplement: Figure S2 — Immunofluorescent staining of Cx43 in Cx43-EGFP LLC-PK1 cells. Cx43-EGFP cells were exposed to 35 μM CdCl2 for the indicated time and stained for Cx43 (red) and DAPI (nuclei; blue). Immuofluorescent images of EGFP (green), Cx43 (red) and nuclei (blue) were captured using a CCD camera attached to the IF microscope. The merged images of Cx43 and EGFP (yellow) are shown in the right panel. Note the complete overlapping of Cx43 and EGFP (yellow) and the increased intensity of perinuclear Cx43 following Cd2+ stimulation. (TIF) [file pone.0058057.s002.tif]

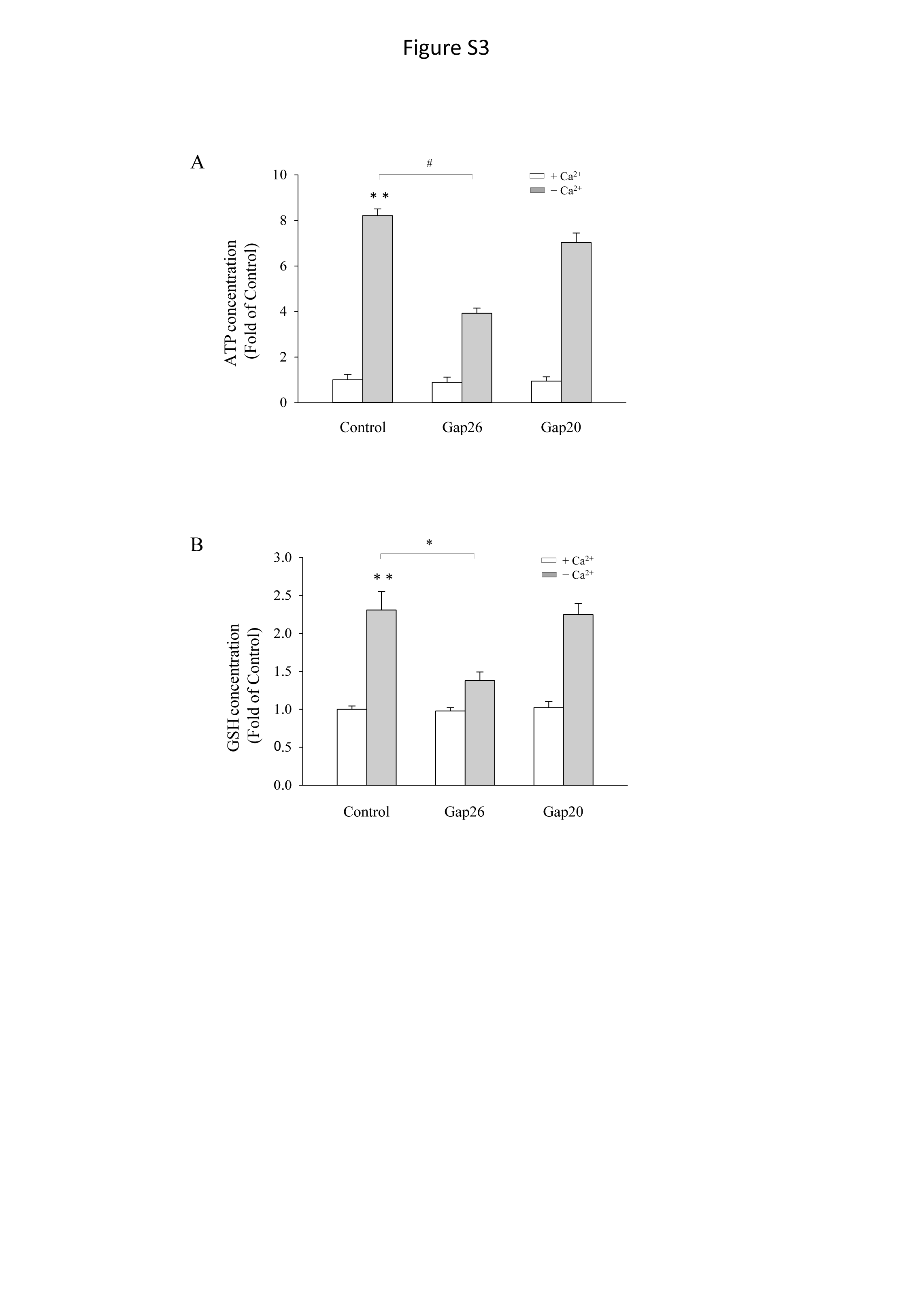

Supplement: Figure S3 — Effect of Cx43 mimetic peptides on calcium deprivation-triggered release of ATP and GSH in Cx43-LLC-PK1 cells. Cx43-LLC-PK1 cells were pretreated with Cx mimetic peptides Gap20 and Gap26 at the concentration of 100 μM for 1 h, and then changed to calcium-free medium containing the same amount of peptides for additional 15 min. ATP (A) and GSH (B) concentration in culture medium was measured. The data were expressed as the fold induction against untreated control (mean ± S.E., n = 4). # p<0.01, * p<0.05 versus Cd2+ alone, and ** <0.01 versus untreated control. (TIF) [file pone.0058057.s003.tif]
